# An Integrated Machine Learning Approach for Congestive Heart Failure Prediction

**DOI:** 10.3390/diagnostics14070736

**Published:** 2024-03-29

**Authors:** M. Sheetal Singh, Khelchandra Thongam, Prakash Choudhary, P. K. Bhagat

**Affiliations:** 1 Department of Computer Science and Engineering, National Institute of Technology Manipur, Langol, Imphal 795004, Manipur, India; msheetalsingh@live.com (M.S.S.);; 2 Department of Computer Science and Engineering, Central University of Rajasthan, Tehsil Kishangarh, Ajmer 305817, Rajasthan, India; 3 Department of Computer Engineering and Applications, GLA University, Mathura 281406, Uttar Pradesh, India; pk.bhagat@gla.ac.in

**Keywords:** CHF prediction, CHS, DNN, KNN, C4.5, imputation

## Abstract

Congestive heart failure (CHF) is one of the primary sources of mortality and morbidity among the global population. Over 26 million individuals globally are affected by heart disease, and its prevalence is rising by 2% yearly. With advances in healthcare technologies, if we predict CHF in the early stages, one of the leading global mortality factors can be reduced. Therefore, the main objective of this study is to use machine learning applications to enhance the diagnosis of CHF and to reduce the cost of diagnosis by employing minimum features to forecast the possibility of a CHF occurring. We employ a deep neural network (DNN) classifier for CHF classification and compare the performance of DNN with various machine learning classifiers. In this research, we use a very challenging dataset, called the Cardiovascular Health Study (CHS) dataset, and a unique pre-processing technique by integrating C4.5 and K-nearest neighbor (KNN). While the C4.5 technique is used to find significant features and remove the outlier data from the dataset, the KNN algorithm is employed for missing data imputation. For classification, we compare six state-of-the-art machine learning (ML) algorithms (KNN, logistic regression (LR), naive Bayes (NB), random forest (RF), support vector machine (SVM), and decision tree (DT)) with DNN. To evaluate the performance, we use seven statistical measurements (i.e., accuracy, specificity, sensitivity, F1-score, precision, Matthew’s correlation coefficient, and false positive rate). Overall, our results reflect our proposed integrated approach, which outperformed other machine learning algorithms in terms of CHF prediction, reducing patient expenses by reducing the number of medical tests. The proposed model obtained 97.03% F1-score, 95.30% accuracy, 96.49% sensitivity, and 97.58% precision.

## 1. Introduction

The main organ of the human body is the heart. If it is not functioning properly, an infection can spread throughout the body, and the sickness typically leads to death. It regulates our body temperature, blood pressure, and many other crucial health-related factors, like blood oxygen levels [1]. Congestive heart failure (CHF) is a condition in which the heart is not able to pump the required amount of blood to the body. In this condition, blood accumulates in the heart, and pulmonary circulation causes congestion, and thus, fluid builds up around the heart and lungs, further causing heart failure. The main cause of CHF is decreased preload, decreased contractility of myocardium, and increased afterload. Valvular dysfunction, aneurysm, or plaque may be the reason behind this. The main types of CHF include left-sided heart failure and right-sided heart failure, which may be systolic or diastolic depending upon the complications [1].

Heart disease is the main reason for hospitalizations in elderly individuals over 65 years of age [2]. The American Heart Association states that over 50% of all American adults (i.e., 121.5 million) have one or more cardiovascular diseases [3]. According to World Health Organization (WHO) reports, heart disease causes more than 15 million fatalities worldwide, which accounts for around 30% of all deaths worldwide. If unmitigated, it is predicted that, in 2030, there will be approximately 22 million deaths globally. Some of the major causes that can develop into heart disease include diabetes, high cholesterol, physical inactivity, high blood pressure, family genetics, excessive use of alcohol, unhealthy diet, tobacco, and many other factors [4,5,6,7]. Sometimes, different genders may experience different CHF symptoms. For instance, a male patient can more possibly experience pain in the chest. In contrast, a female patient may experience additional symptoms, such as nausea, acute exhaustion, and shortness of breath, along with chest pain [8]. These uncertainties can be reduced by adopting a healthy way of living, such as consuming less salt, ingesting vegetables and fruits, exercising regularly, and giving up alcohol and cigarettes. This may help reduce the chance of developing heart disease over time.

Since heart disease has a very complex nature, it involves cautious administration. Failure to do so can cause damage to the heart or result in an untimely demise. In more than 40% of cases, heart attacks occur suddenly. Despite the best medical treatment, such events are often fatal and very severe, making it impossible to save a life. The prediction of CHF in its early stages can help to find a cure and save many lives. To diagnose CHF, available diagnostic methods are X-ray, B.N.P. (brain natriuretic peptide), echocardiogram, and cardiac catheterization. Manual methods for diagnosing CHF include the identification of jugular vein distention in the neck, the swelling of extremities, systemic congestion, and maybe liver cirrhosis. The quality of service (QoS) in the healthcare industry, which provides accurate and prompt disease diagnosis and competent patient care, is experiencing serious challenges. A specially trained artificial intelligence (AI) model can be used as an aid to medical professionals in diagnosis. This can speed up the diagnosis process and reduce their workload. This can also save the precious time for medical professionals [9]. In this context, machine learning (ML) algorithms are effective and reliable resources for identifying and classifying patients with and without CHF. Therefore, using machine learning and data mining techniques, researchers have created a wide range of automatic systems for diagnosis [10,11]. The current approaches have some limitations, such as hefty data produced by CT scans or MRI scans and space complexity, where it is challenging to develop a solution to distinguish between heart disease and stroke. Heart disease prediction is considered to be challenging; however, with the development of ML algorithms, it is now an important subject. Employing AI helps both patients and doctors to save lives by undertaking treatment as soon as possible using electronic health records (EHRs), which are useful in this modern period for both clinical and research purposes by increasing productivity and effectiveness in healthcare. To make the prediction system more exact and accurate, there is now an urge to increase accuracy.

By minimizing the prediction error and actual outcomes, different ML techniques have been employed to comprehend the non-linear relationship between various parameters and complexity [10]. The outlook of clinical research and data mining techniques are applied to identify several types of anabolism syndromes. Classification and data mining both serve a crucial function in data analysis and heart disease prediction. Furthermore, we have observed the usage of decision trees to identify events related to heart disease [12]. However, when it comes to machine learning, imbalance and outlier data can emerge and affect the effectiveness of the prediction model. Earlier research has shown that the outlier data can be identified and eliminated by using density-based spatial clustering of applications with noise-based methods [13], and to balance the distribution of data, a hybrid synthetic minority over-sampling technique-edited nearest neighbor (SMOTE-ENN) is used to significantly improve the performances of the prediction models [14]. In order to overcome these problems, we propose an effective hybrid model for CHF prediction, where we employ the C4.5 algorithm for feature selection from an inconsistent and noisy dataset, the KNN algorithm for missing data imputation, and DNN to predict CHF. In this work, various observations were made to develop a prediction model employing a unique methodology. These integrated new techniques are often referred to as hybrid methods [15].

**Figure 1:**
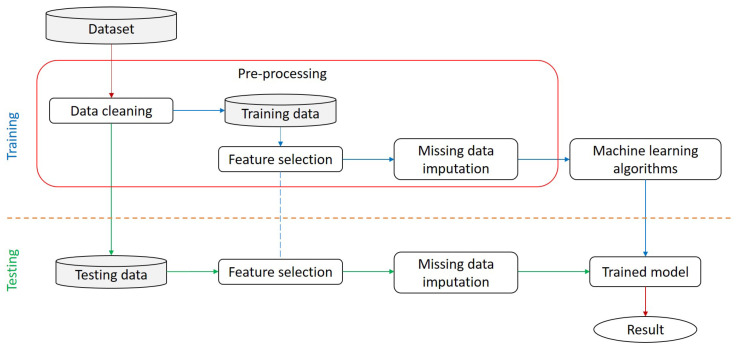
A block diagram to depict the generalized stages of the proposed model.

**Figure 2:**
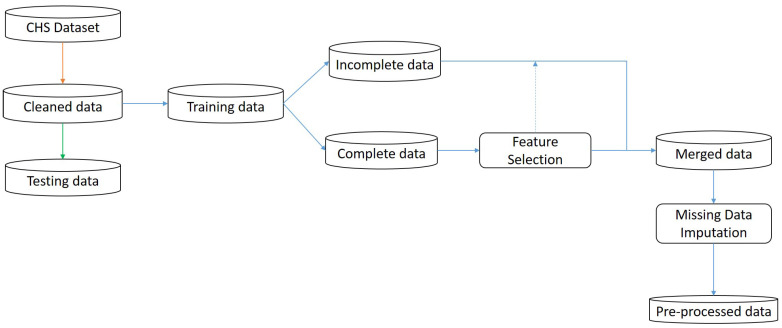
The stage-by-stage process performed on the dataset during pre-processing.

In this work, our goal is to help the physician diagnose CHF in its early stage by identifying the optimal feature from all the features present in the dataset, which improves the overall performance of CHF prediction and minimizes the number of tests to be performed by healthcare. A block diagram of the proposed model is shown in Figure 1. As we can see in Figure 1, first, the whole dataset is cleaned, where all the duplicate and inconsistent records are removed, and then, we split the remaining data into training and testing sets in the ratio of 70:30, respectively. The ratio of training to testing data is a balance between providing sufficient data for model learning and ensuring a robust evaluation of unseen instances. And, we can see in Figure 2 that in feature selection, we further split the training set into complete and incomplete sets, in which features are selected from the complete set to prevent the model from selecting irrelevant or suboptimal features that affect the model’s performance. Figure 1 shows the general block diagram of our proposed model, and in Figure 2, we can see all the steps involved in the preprocessing. The CHS dataset was used to build our models and employ seven machine learning models: Naive NB, RF, SVM, KNN, DT, and deep neural network (DNN). After comparing all seven models, DNN performed better than other models with our proposed pre-processing technique. The main goal of this research is to design an effective CHF prediction model using a DNN classifier with improved and enhanced accuracy. The overview of our work’s contributions is as follows: (a) We provide an understanding of the various risk factors for CHF prediction. We examine the numerous factors found in patient records to determine the most crucial factors required for CHF prediction, reducing the need for physical examinations, promptly identifying new patients, and shortening the length of the diagnostic process. (b) We analyze and evaluate the most effective method for predicting the development of CHF by benchmarking widely used machine learning models for heart disease prediction. (c) Our model helps enhance CHF diagnosis using a minimum feature that reduces the number of tests and medical bills. (d) Additionally, the use of digitized healthcare offers a multitude of prospects for mitigating human errors, enhancing therapeutic outcomes, monitoring longitudinal data, and so forth.

The remainder of the paper is organized into different sections as follows. Section 2 presents the background of the proposed problem along with preliminaries. Section 3 discusses a brief description of existing methods and available models. Section 4 discusses the proposed methodology, dataset description, and pre-processing methodology. Section 5 and Section 6 present state-of-the-art classification models used for the experiment and the performance evaluation metrics, respectively. The experimental setup, analysis of results, and comparison with earlier works are presented in Section 7. Finally, Section 8 represents the conclusion and future directions.

## 2. Background and Preliminaries

Congestive heart failure takes place when the heart is not able to distribute blood adequately to meet a body’s requirements [16]. The death rate from heart failure can range from 5% to 75% every year. Most of the risk factors for heart failure comprise high blood pressure, a previous heart attack, being overweight, smoking, abusing alcohol, lack of vitamins, lack of sleep, consuming unhealthy foods (such as animal fats), and laziness [17]. Heart failure is more common in people over 65, those who are overweight, and those who have already experienced heart failure.

The available method for heart disease prediction is very costly, and low-income families cannot afford it. The prediction of CHF may facilitate decisions about specific medications or devices. Current risk prediction techniques, however, have very moderate success rates, most likely as a result of the fact that they were developed using statistical analytic techniques that miss predictive data with multi-dimensional correlations in huge collections of data. Data mining techniques play an important role in finding hidden information from a collection of prior archives for decision-making in the future. Almost every area of life, including business, engineering, medicine, and education, uses data mining.

The world today makes use of electronic health records (EHRs) for both clinical and research purposes where data mining techniques are used. And, studies have claimed that there are several risk factors (high cholesterol, diabetes, alcohol consumption, smoking, and insufficient exercise) that cause heart disease [5]. The usage of various techniques and algorithms has been observed in much research to identify events related to heart disease. Several observations were made in this work in order to develop a prediction model using an integrated methodology [12]. Here, we used the Cardiovascular Health Study (CHS) dataset, which is a challenging dataset with vast inconsistencies and missing values. There are many challenges in this dataset, like the many attributes that are irrelevant to the problem we are trying to solve, such as seeing well enough to recognize a person, waking up at night, having a grandchild born in the past 6 months, etc.; data entry errors; noisy data; and missing values. There are more than 30% of data missing; some of the reasons are participant refusal, the inability to answer certain questions, or death. Manual input error is one of the problems because it leads to some missing data but can also add to data inconsistency.

To make the dataset suitable for the classification algorithm, we applied an integrated method by combining the decision tree with KNN in pre-processing, where the decision tree serves as a feature selection algorithm and KNN as a missing data imputation algorithm. Initially, we separated CHF records from the raw data and split them into two subsets (complete and incomplete). Then, DT C4.5 was applied to the complete set to calculate the gain ratio value for each attribute to rank important features in the dataset, select top features, and discard the remaining features with a lower information gain ratio. For the missing values present in the incomplete set, we merged the complete and incomplete sets with respect to the selected attributes. Then, KNN was used to replace the missing value with the nearest possible value within the dataset. After the pre-processing of raw data, the dataset was cleaned and ready for classification without any missing and inconsistent values. The pre-processed data were used as input for six standard ML algorithms (KNN, LR, NB, RF, SVM, DT), and a DNN, and the performance was evaluated using a 10-fold cross-validation method. The result shows that the integrated pre-processing method with the DNN as a classifier outperformed other standard ML classifiers with maximum accuracy. After analyzing the outcomes, this model can be used to predict CHF and can help improve the condition by adopting specific medications or devices before it is too late. For further improvement of the model performance, different ML techniques can be integrated or combined in new possible ways to predict different diseases using various datasets. In the future, the model can be a reliable and cost-effective tool against expensive procedures to predict disease. A summary of key notations used throughout this article is given in Table 1.

## 3. Related Work

The non-linear Cleveland heart disease dataset was utilized in [18] for predicting heart disease using the properties of random forests with minor modifications. Every attribute with incomplete data was taken, and the median of that attribute was used to fill in the incomplete data values. The dataset for heart disease was cleaned by removing any missing values. They achieved more accurate heart disease predictions by employing the RF classification technique when the attributes were well-defined. By employing 10-fold cross-validation, the accuracy of random forest was verified after 303 instances of data were trained. The suggested model outperformed the competing models by achieving maximum accuracy.

A heart sound-based technique for detecting CHF was presented by Gjoreski et al. in [19]. They concentrated on using the analysis of cardiac sound recordings to determine the CHF condition. The approach combined the traditional ML approach and the end-to-end deep learning (DL) approach. A spectro-temporal representation of the signal was used as input for the DL algorithm to learn, whereas traditional ML used expert characteristics to learn. As many as 947 publicly accessible individual recordings and one CHF dataset were gathered, and the proposed method was evaluated. The ML models were created using 15 features to differentiate between CHF phases.

Plati et al. proposed a model using different machine learning classifiers (Bayes network (BN), decision tree, SVM, logistic model tree (LMT), RF, KNN, aive Bayes (NB), and rotation forest (ROT)) to diagnose heart failure on 422 subjects with 10-fold cross-validation for the evaluation [20]. A total of 73 female and 154 male subjects made up the HF sample, whereas 106 male and 151 female subjects made up the non-HF dataset. LMT and ROT performed better than any other classifiers after being compared.

Gjoreski et al. presented a method by stacking different ML classifiers for detecting CHF from the sounds of the heart [21]. A professional digital stethoscope was used to capture the sounds. They used 152 distinct heart sounds from 122 different people in all. In the segment-based ML phase, experiments were conducted using combinations of various types of models, ranging from specific techniques to an aggregate of seven techniques—NB, Bagging, RF, SVM, KNN, Boosting, and J48. For the evaluation, these models employed the LOSO cross-validation method. An accuracy of 96% was achieved by the experimental approach, which revealed encouraging findings.

A heart failure risk assessment prediction model was proposed by Aljaaf et al. using the C4.5 classifier on multiple levels, where heart failure is categorized into five heart failure risk levels, namely, high-risk, low-risk, extreme-risk, moderate-risk, and no-risk [22]. This study made use of heart disease information from the Cleveland Clinic Foundation. Three supplementary features—physical activity, smoking, and obesity—that significantly increase the risk of getting heart failure were added to the dataset as part of the study’s focus on improving dataset features. Ten-fold cross-validation was employed to measure performance. With 95.5% specificities, 86.5% sensitivity, and 86.53% accuracy, the prediction model outperforms several other models.

By integrating the computing capacity of several ML techniques, such as RF, KNN, DT, and SVM, and using Cleveland Heart Disease Dataset, Srivastava et al. proposed a revolutionary way to diagnose heart disease [23]. The result showed that KNN provided the maximum accuracy of any method. After training, the model was used to create packages that could be uploaded to a web server, and a web interface was created that allowed users to enter attributes and see the results.

Awan et al. proposed a model using the UCI dataset for the prediction of heart disease. For the prediction of heart disease, the KDD model and neural network approaches were used, and the results before and after PCA were compared [24]. Using the PCA algorithm, the data were pre-processed for training before being input into the ANN. Using principal component analysis (PCA), the accuracy rate increased from 94.7% to 97.7%. This is how the accuracy is computed and represented.

A unique approach called Hybrid Random Forest with Linear Model (HRFLM) was presented by Mohan et al. in [25] for accurate heart disease predictions utilizing heart rate time series. The Cleveland UCI repository is where the dataset was gathered. The HRFLM method bases feature selection on DT entropy, and feature selection and modeling are repeated for different combinations of attributes to maximize the performance in cardiovascular disease prediction. Three heart disease prediction models (RF, LM, and DT) were employed as classification, and a confusion matrix was used for the evaluation of the model. The characteristics of the suggested hybrid linear method (LM) and random forest (RF) technique were combined. Comparing the HRFLM classification method to other approaches, it had the highest accuracy.

Shaji et al. used data mining technologies in their research to predict and enhance the performance of the diagnosis of heart disease [26]. By interacting with patients and gathering data from discharge summaries of the patients, the data were gathered from Jubilee Mission Hospital in Thrissur. Twenty attributes were collected from roughly 2200 patients in total, and they were entered into an Excel file. Then, classification was performed using data mining techniques including ANN, RF, and KNN, where ANN delivered the best outcome and maximum accuracy.

To predict cardiac illness, Sharanyaa et al. proposed a hybrid strategy that combined the virtues of fuzzy logic and the KNN algorithm [27]. Various classification methods used clinical data values, and the algorithms KNN, SVM, DT, and RF were used to forecast heart disease. A total of 13 features and four classifiers were used to forecast disease and maximize the accuracy of the model. Compared to other methods, the KNN classification outperformed with the highest accuracy.

Singh et al. used the Cleveland and Framingham dataset for the heart disease prediction model to increase the performance accuracy [28]. Random forest was combined with logistic regression since it is a very robust model that provides superior accuracy. Following a comparison of the outcomes of different ML and DL models, the idea of a hybrid technique utilizing weighted average aided in heart disease prediction for better performance.

To improve the accuracy of predictions, Fitriyani et al. developed a system that supports clinical decisions by integrating SMOTE-ENN, Density-based Spatial Clustering of Applications with Noise (DBSCAN), and XGBoost-based MLA [29]. This system can be utilized to diagnose the individual’s heart state earlier. The outlier data were found and removed using DBSCAN, the uneven training dataset was balanced using SMOTE-ENN, and the prediction model was learned and created using XGBoost MLA. The model was developed using Statlog and Cleveland, two publicly accessible datasets. The proposed method outperformed other methods and past research after a performance comparison with other categorization models.

By using nine classical models—Gaussian naive Bayes classifier (G-NB), adaptive boosting classifier (AdaBoost), gradient boosting classifier (GBM), SVM, ETC, SGD classifier, DT, LR, and RF, Ishaq et al. showed that effective data mining approaches on significant features can increase the accuracy in cardiovascular patients [30]. To address the imbalance in class, they used SMOTE. Furthermore, RF was employed to select the highest-ranking features used to train machine learning models.

Alaa Khaleel Faieq et al. [31] employed ML techniques (SVM and ANN) in the prediction of heart disease using a UCI ML repositories database, which contained medical information of 170 subjects. Here, SVM outperformed ANN with an accuracy of 89.10% in heart disease prediction.

Abdul Saboor et al. [32] proposed a machine learning classifier with different state-of-the-art models ((RF, XGBoost, CART, SVM, multinomial naïve Bayes (MNB), LR, linear discriminant analysis (LDA), AdaBoost classifier (AB), and ET) for heart disease prediction. The author used the Cleveland heart disease dataset for testing.

The performance of the proposed SVM outperforms the other classifiers. Gunjan Gupta et al. [33] designed a model KNN for heart disease prediction using a dataset collected from Kaggle. They also compared the model with different classifiers, like DT, KNN, ANN, NB, and RF. The outcome of the comparison showed that with the value of k = 5, the proposed model with KNN had the highest accuracy.

Umarani Nagavelli et al. [34] proposed a tool using XGBoost to improve the accuracy of heart disease diagnosis prediction. They compared four types of machine learning (ML) models. From the abovementioned table and graphs, it is clear that the accuracy parameter is high in the XGBoost algorithm-based heart disease detection.

Using an RF algorithm, Vien T. Truong et al. [35] determined the diagnosis of congenital heart disease (CHD) using postnatal echocardiography. They also demonstrated greater sensitivity in prenatal CHD screening with very excellent performance.

All previous research has focused on classifying heart failure from non-heart failure, using various features, datasets, and methods. The comparative analysis of earlier work in this study helps to identify the effectiveness and weaknesses of previously proposed ML techniques for the diagnosis of heart disease. A summary of the related work is presented in Table 2. Most of the previous research used small datasets (less than 300 samples), which resulted in fewer observations in test data and could lead to overfitting. Several optimization techniques have been used in their work to enhance a number of measures, namely precision, recall, and accuracy. One of the primary aims of our work is to evaluate various ML algorithms to analyze which method is best for CHF prediction. We anticipate that our prediction model performs better than the approaches used in earlier research and state-of-the-art models.

## 4. Materials and Methods

### 4.1. Dataset

In this work, the CHF data was collected from the BioLINCC CHS data package [36]. The Cardiovascular Health Study (CHS) is a comprehensive investigation conducted on a population level, focusing on the longitudinal examination of coronary heart disease and stroke occurrences among individuals who are 65 years of age or older [37]. The dataset contains the medical records of 5888 participants from four regions of the United States who underwent thorough clinic evaluations to identify signs of underlying cardiovascular disease. Out of 5888 participants, 2495 were men, and 3393 were women, with about 416 attributes. The study enrolled people who met the eligibility criteria and provided informed consent. These participants were then administered standard questionnaires to gather information on lifestyle habits, family history, medication usage, and medical history, which encompassed hospitalizations and previous diagnoses of cardiovascular and cerebrovascular illnesses. The primary aim of this study was to discover the parameters associated with the initiation and progression of coronary heart disease and stroke [38]. There are many challenges in this dataset, like the many attributes that are irrelevant to the problem we are trying to solve, such as seeing well enough to recognize a person, waking up at night, having a grandchild born in the past 6 months, etc.; data entry errors, noisy data, and missing values. In addition, the data entry is conducted by humans via telephone calls or by keying in a written data form or printed source. In these settings, data are often corrupted upon entry because of typographic errors or a misunderstanding of the data source. Mainly, more than 30% data were missing; some of the reasons include participant refusal, inability to answer certain questions, or death. Manual input error was one of the problems because it would lead to some missing data but could also lead to data inconsistency. The prevalence of underreporting was also observed frequently. The prevalence of self-reported CHF was found to be 73.3% among men and 76.6% among women. Similarly, the occurrence of stroke was reported by 59.6% of men and 53.8% of women. Additionally, transient ischemic attack was self-reported by 41.5% of men and 37.0% of women [39]. The dataset contained details of more than ten cardiovascular-related diseases, and more than 50% of data were not related to CHF or were missing, which made the dataset more challenging for the prediction of CHF.

### 4.2. Pre-Processing

Pre-processing data is a fundamental stage that directly affects the outcomes of the classification algorithm. The CHS dataset is a challenging dataset corrupted with duplicates, noise, incomplete information, inconsistencies, and missing values. Applying a classification algorithm directly to this type of dataset would not give a good result or would fail to predict effectively. In pre-processing, the unstable data are processed into a useful and understandable format suitable for the classification model. As we can see in Figure 2, in this work, we applied a unique combination of steps in pre-processing. Initially, we cleaned the raw data (CHS); then, we separated the testing set and further pre-processed the training set by splitting it into complete and incomplete sets. Then, we selected the best feature from the complete set using the C4.5 algorithm, and the same feature was also selected from the incomplete set. Then, we merged both complete and incomplete sets to become one complete dataset. Hence, we merged an incomplete set, which consisted of missing values. To handle the missing values, we filled the missing values using the KNN imputation technique. The detail stages in pre-processing are as follows:

- Cleaning;
- Splitting;
- Feature selection;
- Missing data imputation.

#### 4.2.1. Cleaning

Data cleaning in the pre-processing is one of the crucial steps that improve the quality of data and help in analysis. In data cleaning, we first removed all the non-CHF patient records (i.e., 3265 records, which are related to other cardiovascular diseases). Furthermore, to reduce the inconsistencies and errors, redundancies and attributes with more than 30% missing data were removed (i.e., 197 attributes were removed). Imputing a considerable number of missing data, especially when the missingness exceeds 30%, introduces ethical questions in the framework of healthcare. Inaccurate imputations may result in poor predictions, which may have an effect on patient care and assessment. The remaining 219 attributes contained general demographic information, physical examination, medical history, CHF risk factors, habits, laboratory data, and other diseases. Then, we selected the patient’s records related to CHF, and records with no disease were selected for classification. After cleaning the raw dataset, the remaining 2623 records (2043 diagnosed with CHF and 580 with no CHF) were divided into a ratio of 70:30 between the training and testing sets, and the training set was used in the following steps.

#### 4.2.2. Splitting

Furthermore, the training data were divided into two sets, a complete set with no missing data and an incomplete set with missing data. Our goal with splitting was to select the optimal features from the complete set without any missing data, and we also selected the same set of features from the incomplete set. Then, we merged the complete and incomplete sets after feature selection and performed missing data imputation.

#### 4.2.3. Feature Selection

**Figure 3:**
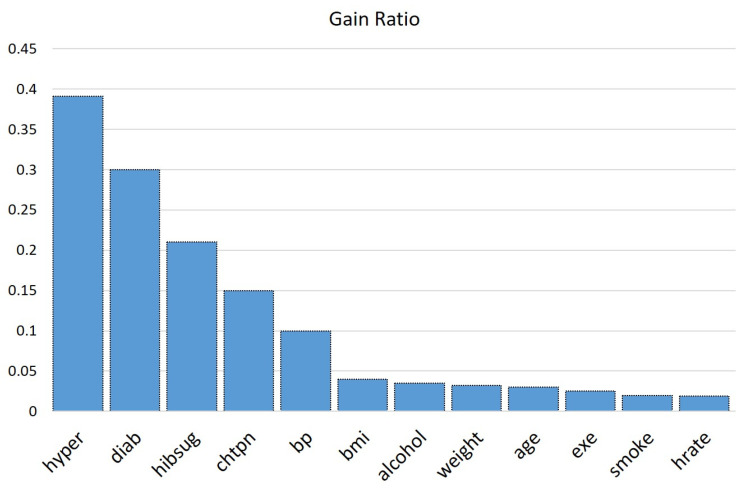
Visual representation of the Gain ratio of each feature selected for further processing.

After splitting the training data into two sets (complete set and incomplete set), feature selection was conducted on the complete set part. To avoid overfitting, we employed a feature selection process that further enhanced the performance of a classification algorithm and reduced the computational time by selecting the subset of the dataset that provided better interpretability. After understanding other feature selection methods with respect to the characteristics of the CHS dataset, we selected C4.5 [40] for feature selection because of its capacity to quantify the efficiency of each attribute in identifying the data by calculating the information gain ratio of the attributes. This not only underscores the significance of specific characteristics but also directs the process of selection by highlighting their effect on the overall classification accuracy. In addition, the selected feature selection method takes the duplication of attributes into account, resulting in a more comprehensive evaluation. This guarantees that the chosen features not only provide a meaningful contribution to the task but also prevent unnecessary duplication, hence improving the efficiency of the selected subset. This makes it especially suitable for our integrated hybrid model. With the help of data visualization, we could unfold the concealed patterns that were present inside the dataset by showing the features’ characteristics. We selected the 12 best attributes (shown in Table 3) with high correlation with the class. We analyzed and experimented with various attribute sets (top 8 attributes to top 25 attributes), and the attribute set with top 12 attributes gave the best result. Figure 3 represents the information gain ratio of the features selected by the C4.5 algorithm. A detailed discussion about selected features is presented in Section 7.4. The feature selection steps are shown below:

- Step 1. For all the features, calculate the entropy from the complete dataset [41].
- Step 2. Use calculated entropy to determine information gain value [41,42].
- Step 3. Calculate the information gain ratio [41,42].
- Step 4. Sort the attributes concerning the information gain ratio [43].
- Step 5. Select the top 12 attributes [43].

#### 4.2.4. Missing Data Imputation

In this stage, we imputed the missing data with a substitute value (known value from the dataset) to retain most of the information of the dataset. If we removed all the missing data from the dataset, the dimensions of the dataset would be reduced, which would lead to an incorrect analysis, or if we kept the missing data, distortions in the variable distribution could result. With respect to the characteristics of the dataset and nature of the missing data, we sought an imputation technique that preserves local patterns and relationships in the data, which is crucial for upholding the clinical relevance, quality, and understanding of the data. These criteria are of utmost importance in healthcare decision-making and research. Other imputation methods, like mean, median, mode, and hot deck, ignore the relationship in the data. Additionally, KNN is robust against outliers and less computationally demanding. In our work, we used the KNN technique for missing data imputation. The technique identifies ‘k’ samples that are close or similar in the dataset by calculating the Euclidean distance between the samples in the space. The value of the missing data points can then be evaluated using these ‘k’ samples. The mean score of the dataset of the ‘k’ neighbors is used to impute the missing values of each sample [44]. In our experiment, the value of ‘k’ was 5.

## 5. Classification

For classification, various ML classification algorithms were employed first to train and validate using the pre-processed training dataset, and the performance was evaluated using the testing set for the prediction of CHF. The classifiers were the LR, RF, SVM, KNN, DT, and DNN algorithms.

### 5.1. Decision Tree

The DT approach is a simple, easy, and powerful supervised learning method used for regression and classification [45]. The J48 algorithm is used for this system [46,47].

### 5.2. Support Vector Machine

SVM is a supervised learning model based on mathematics. It is a linear model for problems involving regression and classification [25,48].

### 5.3. K-Nearest Neighbor

KNN, also known as case-based reasoning, mostly utilized for classification and regression, is one of the simplest supervised learning techniques [23]. In this work, we employed an efficient variant of KNN, called Ensemble Centroid Displacement-based KNN (ECDNN), which leverages the homogeneity of the nearest neighbors of test instances. ECDNN displays more effective and robust results when compared to those of other variants of KNN [49,50].

### 5.4. Random Forest

One of the most efficient ensemble-supervised classification algorithms is the RF technique. This algorithm has been employed in probability and prediction [30].

### 5.5. Logistic Regression

LR is a probability-based supervised learning algorithm typically assigned to classification problems. LR is a fast and effective solution to linear and binary classification problems [30,51].

### 5.6. Deep Neural Network

A DNN is a machine learning architecture in which multiple neural networks are layered to form a layer of an interconnected network [52]. The depth of the network is determined by how many layers of neurons there are between the input and output units. The architecture of a DNN, consisting of numerous hidden layers, is deliberately created to acquire hierarchical representations, allowing it to autonomously identify and simulate complex patterns. DNN, also known as feed-forward DNN (FF-DNN), often flows in a single direction because of the network’s deep structure. The DNN has several issues during training (such as overfitting and computation time), but once trained, it can simulate intricate non-linear relationships. DNNs are often used for classification, and it has been shown that they often outperform some popular classifiers, like SVM [53]. It is highly challenging for a DNN model to outperform human intellect, even though it attempts to simulate human brain activity. A DNN model has, nevertheless, occasionally outperformed human intelligence. But, there have also been several cases where these models have been deceived [54]. The progress in deep learning approaches, encompassing enhanced designs and algorithmic optimization, enhances the attractiveness of DNNs in this setting. In this study, for a complicated and non-linear pattern dataset, we employed a DNN model that could accurately represent complex, detailed relationships. Furthermore, the dataset consisted of a wide range of features, encompassing both numerical and categorical data. The ability of the DNN model to handle variations in features was crucial in these situations, enabling the model to analyze and understand complex connections between multiple types of information. Moreover, a DNN has the capacity to deal with non-linearity using activation functions, such as ReLU, which makes it highly suitable for encapsulating intricate, non-linear relationships underlying the dataset. To design our DNN model to predict CHF, we used four hidden layers and ReLU as an activation function to create a DNN model as a classification algorithm for CHF prediction. The stochastic gradient descent algorithm was used as an optimization algorithm to train the network. The Keras toolbox was used to create the DNN model, with TensorFlow serving as the backend. The details of parameters used with the proposed DNN model are shown in Table 4.

## 6. Evaluation Metrices

In this study, the performance differences in classifiers were examined using a confusion matrix with regards to precision, accuracy, false positive rate (FPR), sensitivity, MCC, F1-score, and specificity [55]. The performance of the proposed model was measured using Equations (1)–(7).

(1) $$Accuracy=\frac{TP+TN}{TN+FN+FP+TP}$$

(2) $$F1\text{-}score=\frac{2TP}{2TP+FP+FN}$$

(3) $$Specificity=\frac{TN}{TN+FP}$$

(4) $$Sensitivity=\frac{TP}{TP+FN}$$

(5) $$Precision=\frac{TP}{TP+FP}$$

(6) $$FPR=\frac{FP}{FP+TN}$$

(7) $$MCC=\frac{\left(TN\times TP)-(FN\times FP\right)}{\sqrt{\left(TP+FP)(TP+FN)(TN+FP)(TN+FN\right)}}$$

## 7. Experimental Results Analysis

The experimental methodology and findings from all of the CHF prediction experiments are discussed in this section. The dataset included information regarding lifestyle, clinical, and bodily characteristic attributes. Some of these characteristics, like gender, smoking, diabetes, and blood pressure, are binary. After pre-processing, the dataset consisted of 12 best features and no missing values. The pre-processed dataset was utilized for training the machine learning models, which were then evaluated on precision, sensitivity, specificity, accuracy, F1-score, and Matthew’s correlation coefficient. Figure 1 represents the proposed methodology’s flowchart.

### 7.1. Experimental Design

To evaluate the performance of the models, we employed supervised machine learning techniques. To prevent overfitting, the dataset was split into a ratio of 70:30 between the training set and the testing set. This ratio was employed in various works of literature for classification tasks [56]. Different performance evaluation metrics were employed to test the machine learning classifier’s performance. Hardware details are shown in Table 5.

### 7.2. Experimental Results

**Figure 4:**
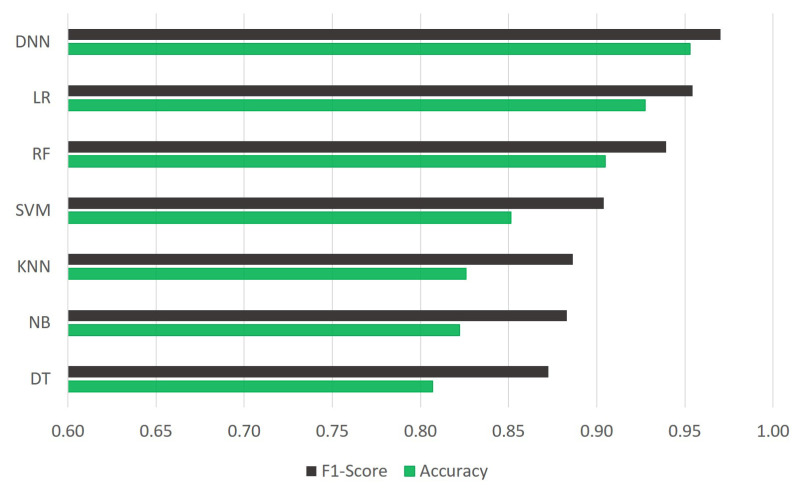
A comparision of F1-score and accuracy obtained with different classifiers.

Early identification of CHF is crucial for individuals at risk in order to prevent the worsening of symptoms and minimize associated risks. This study proposed an integrated automatic CHF diagnosis method using the CHS dataset. In this experiment, machine learning classifiers were employed to evaluate significant features selected by the C4.5 algorithm. The least significant features were eliminated throughout the training and testing of classifiers. By eliminating the least important features, the classifiers improved performance. After eliminating most of the noise from the dataset during data cleaning, feature selection, and missing data imputation, the DNN was applied, and when compared to other ML models, the DNN showed an impressive outcome for improving prediction accuracy. For comparison, we used six MLAs (DT, SVM, KNN, RF, NB, and LR) that are often employed in research and are renowned for accuracy and performance. The F1-scores of all the classifiers are shown in Figure 4. For the validation of every model, we carried out a 10-fold cross-validation on the training set and tested the model performance using the testing set. We carefully considered statistical stability and practical issues when choosing 10-fold cross-validation for our study. This way, we avoided any problems that might come with using fewer folds. The benefit of the 10-fold technique was that it could give a more precise overview of how effectively the model performed. Each iteration’s smaller testing set would make things stabler and give an accurate view of how well the model works across different sets of data. Practically speaking, computational effectiveness is essential. Increasing the number of folds increases processing needs while improving statistical dependability. Practically speaking, very high folds may not be feasible because of significant processing times, particularly with large datasets or complex models. On the other hand, fewer folds may seem preferable for computational simplicity, but there is a cost associated with it. Larger testing sets result from fewer folds, which could increase variability and compromise the accuracy of the model’s assessment. The purposeful selection of 10-fold cross-validation in our study guaranteed a cautious balance. It satisfied both practical limitations and statistical rigor by offering a strong evaluation of model performance despite unnecessarily taxing computing power. To evaluate the performance of the proposed CHF prediction model, we used seven evaluation parameters: precision, accuracy, FPR, TNR, sensitivity/recall, F1-score, and MCC.

### 7.3. Comparison and Performance Analysis

**Figure 5:**
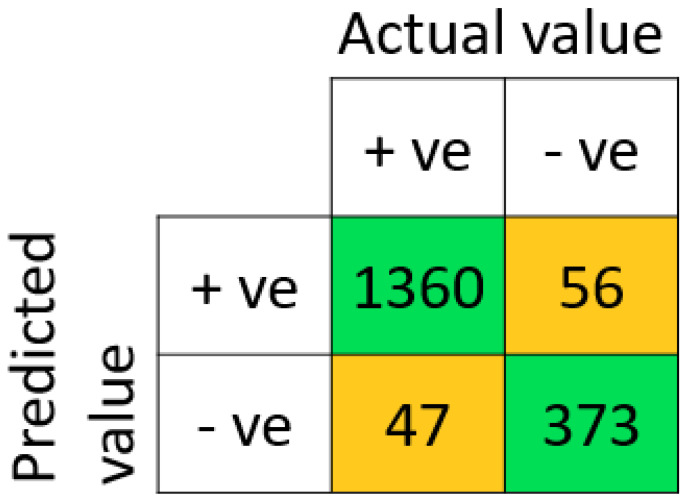
The obtained confusion matrix on the training set with proposed DNN classifier.

**Figure 6:**
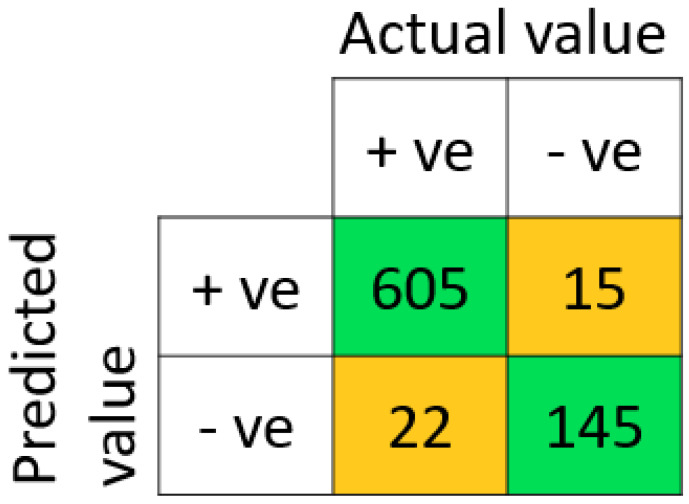
The obtained confusion matrix on the testing set with proposed DNN classifier.

**Figure 7:**
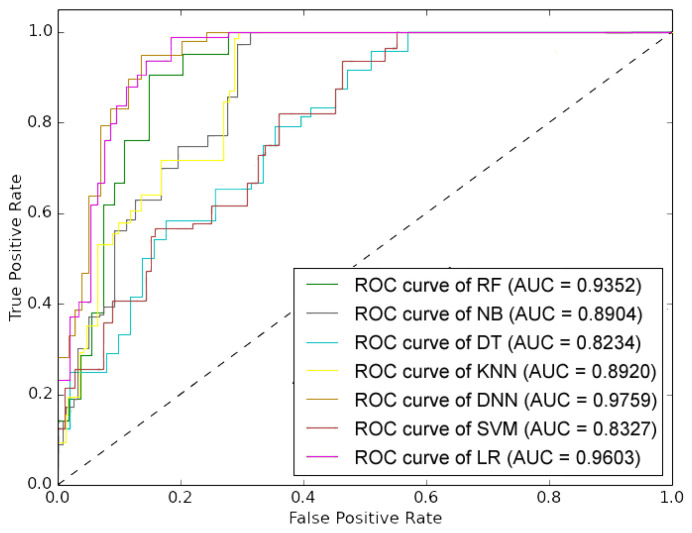
The obtained ROC curve of all the classifiers mentioned in the Table 6.

The performance outcome showed that the proposed method outperformed the other six models by obtaining an F1-score, accuracy, sensitivity, and precision, up to 97.03%, 95.30%, 96.49%, and 97.58%, respectively. The performance outcomes also showed that the suggested model had the highest TNR and lowest FPR in comparison to other ML models (refer Table 6). The proposed methodology achieved a TNR of 90.63% and an FPR of 9.38%. The proposed model’s high TNR and low FPR value showed DNN’s capability to improve prediction accuracy and decrease miss-rate for both positive and negative subjects. In terms of MCC, the DNN showed the maximum 85.75% MCC value, which confirms the efficiency of our proposed model over other state-of-the-art models. The overall performance outcomes are displayed in Table 6, which also reveals that the results of the SVM, KNN, NB, and DT models are low when compared with other classifiers like RF, LR, and DNN. The same can also be observed from the graph in Figure 4. Therefore, this overall analysis (as shown in Table 6) cannot be utilized as the primary evidence to make conclusions about the efficiency of the provided prediction models. However, it can be used to compare the proposed methodologies in general. The obtained confusion matrix for the training set, testing set, and ROC of the model is shown in Figure 5, Figure 6, and Figure 7, respectively.

### 7.4. Discussions

The goal of this study was to create an effective ML pipeline that can predict CHF with reasonable explanations. A secondary objective of this study was to uncover useful risk factors that contribute considerably to the classification output to reduce the diagnosis cost and enhance the performance. The proposed method’s feature selection step can substantially affect the classification performance of feature-based ML methods. Moreover, within each classification model, our approach ultimately employed a fewer-feature subset. The results of the study indicate that a limited set of clinical, biochemical, and demographic parameters are sufficient for establishing a diagnosis of CHF, resulting in reduced time and cost demands. In this research study, we employed simple DL models for predictions of CHF. The purposeful decision to use imbalanced datasets, consisting of 2043 instances of CHF patients and only 580 examples of no CHF, is based on numerous concerns. First and foremost, it is crucial to highlight that the occurrence of CHF in real-world populations appears to be less in comparison to non-CHF instances. The dataset we used accurately represents the basic imbalance, thereby ensuring that our study was in keeping with the normal distribution of occurrences of CHF. The selection of this option was crucial for the clinical significance of our prediction model, as accurately identifying positive cases is of significant importance in healthcare applications. We understand the likelihood of biases that may arise because of the unequal distribution of classes and implemented strategies to successfully mitigate them. The study utilized performance indicators that exhibit reduced sensitivity to imbalance, such as F1-score, recall, and precision. These measures offered a more thorough assessment of the model’s performance, particularly in situations where there was an imbalance in class distributions. We also performed sensitivity analyses to see what happens to the model’s predictions when the imbalance between classes changes. It is clear from these analyses that our method is robust and can work in a wide range of challenging environments. In addition, we conducted a comparison between our model’s performance and that of baseline models and other methodologies. This comparison demonstrates that our chosen methodology successfully utilized the imbalanced data to obtain better outcomes. Ultimately, the utilization of imbalanced datasets was a deliberate and defensible decision in our research. Our methodology guarantees that our predictive model is both of medical importance and capable of addressing the natural challenges resulting from imbalanced datasets. We value the careful evaluation of these factors and encourage any further input to improve the strength and clarity of our research.

The selection of a subset of features was based on a rigorous procedure with the goal of maximizing the model’s performance and improving its understanding. When predicting CHF, the objective is to find factors that have a substantial impact on the forecast and offer distinct information, enabling a more detailed comprehension of the patterns linked to CHF. We selected the 12 best attributes (shown in Table 3) with a high correlation with the class. We analyzed and experimented with various attribute sets (from the top 8 attributes to the top 25 attributes), and the attribute set with the top 12 attributes gave the best result. These features have shown a strong correlation with CHF prediction, encompassing crucial medical data and risk factors that are recognized as key factors in the development of CHF. The choice to include certain features was probably based on past research and knowledge in the field. If earlier research consistently shows that certain parameters are good at predicting CHF, they are likely to be given more weight in the selected features. This approach keeps up with what is already known and makes sure that the model fits with the information we already have about what leads to CHF. Using what we have learned from past studies makes the predictive model more valid and useful in healthcare environments. Also, choosing a limited set of 12 attributes demonstrates a deliberate approach to reducing dimensionality. Keeping track of a smaller group of attributes helps avoid overfitting, especially when working with small datasets. Employing a small group of features in the model makes it simpler, which makes it faster to compute and reduces its susceptibility to capturing irrelevant patterns or noise. This method simplifies the process of converting model predictions into practical information for medical professionals, thereby enhancing the practicality of the model’s predictions in real-life situations.

With the use of several machine learning algorithms and the CHS dataset, the current work significantly adds to the development of such a method for the prediction of CHF. Various models presently in use have experimented with various heart disease datasets. Since there were not many studies on the CHS dataset, we chose these models for comparison. A comparison with these current models had a greater opportunity for a quantitative study of our proposed model, even though different heart disease datasets comprise distinct sets of properties. Our approach cannot be directly compared with those using cardiac sound recordings [19] or heart sound characteristics [21], but it may be contrasted with those using datasets similar to ours [18,22,23,24,25,29,30,31,32,33]. Regarding the related work, many studies [18,22,23,24,25,29,30,31,32,57] have conducted their research on the same dataset (i.e., UCI Cleveland heart disease dataset) with different approaches, which display a range of accuracy from 85.81% to 97.70%. So, we utilized most of the previously applied approaches in our dataset and compared the performance, as shown in Table 6. The results obtained for CHF prediction show that the DNN technique surpassed all other ML approaches in terms of accuracy (95.30%), sensitivity (96.49%), and specificity (90.63%) when compared to other ML approaches.

In our study, we used a moderate number of data and used the same pre-processed data for all the classification models, and the DNN we used in our study was a simple feed-forward DNN. By utilizing a standardized, pre-processed dataset, the experiment guaranteed an equitable and impartial comparison between various methods. Eliminating any potential biases resulting from differences in pre-processing procedures ensured a fair and equal basis for analysis. The simplicity of the DNN enhanced transparency and interpretability, enabling a more comprehensible comprehension of the acquired patterns. The DNN, despite being simple, is highly suitable for effectively training on a moderate-sized dataset, rendering it feasible in circumstances where computational capabilities are factors to be taken into account. The model’s analysis of non-linear relationships in the data corresponds to the complexity of patterns that conventional methods may not adequately represent. Moreover, the uniformity of the complexity of models across algorithms improves the comprehensibility of outcomes, enabling a concentrated examination. The FF DNN’s inherent simplicity facilitates the replication and interpretation processes, hence enhancing the reproducibility and transparency of the study. Overall, these favorable qualities emphasize the FF DNN’s function in offering vital insights regarding its efficiency compared to different algorithms in a just, understandable, and reliable way.

The efficacy of the proposed DNN architecture for diagnosing CHF lies in the complex interaction of many design decisions that collectively improve our model’s capabilities. Incorporating the ReLU activation function into the hidden layers makes the model non-linear. This provides a base for the DNN to understand the CHF data’s complicated, non-linear correlations, enabling it to find trends that conventional linear models could have overlooked. The depth of our model consists of four hidden layers with a constantly decreasing number of nodes (50, 30, 20, 10). The model’s depth enables it to systematically learn insight into features at various levels of abstraction. The optimization approach is a crucial factor, with SGD playing a prominent role. We used SGD with a meticulously adjusted learning rate of 0.001. This selection guarantees an even integration during the training process, which is a vital element in the model’s capacity to efficiently acquire representative features. By using ReLU activation, sparsity is introduced, resulting in improved computing efficiency and an enhanced ability of the model to identify important features, which, consequently, reduces the possibility of overfitting. Furthermore, the model’s adaptability is facilitated by the architecture’s flexibility, which enables the utilization of various node configurations in each layer. This adaptability helps the model to effectively accommodate the many patterns found in CHF data. This flexibility is useful for encapsulating the complex structure of medical data. Therefore, the combination of adaptability, non-linearity, and incorporation of depth highlights the DNN as an effective tool for CHF prediction, offering benefits over baseline models.

### 7.5. Limitation

One of the constraints of our work is the lack of appropriate input data and their strong association. The majority of the features in the EHR dataset do not add any new information to the original feature space because of their significant correlation with one another. As a result of the sparse data with high levels of inconsistency, we were unable to train deep learning architectures, which are superior to traditional ML methods in terms of effectiveness. Additionally, the way medical institutions store their information increases the effort for researchers because they contain a number of undesirable traits that have nothing to do with CHF. Therefore, a significant amount of processing time can be saved if the institute maintains patient information that only includes selected properties that are essential for CHF prediction instead of storing and preserving all aspects. As part of our next work, we also intend to independently validate our recommended method and further examine its efficacy before using deeper DL frameworks. We will also enhance the performance of the DL and ML approaches by collecting additional characteristics and parameters.

## 8. Conclusions and Future Work

Early detection of congestive heart failure is crucial for individuals at risk, as it allows for timely intervention to prevent the worsening of symptoms and mitigate associated risks. This study employed limited clinical features through feature selection, hence reducing the necessity for diagnostic testing. Unlike previous studies, in this study, we proposed a robust CHF prediction model by integrating C4.5, KNN, and DNN machine learning algorithms to optimize prediction accuracy. We also dealt with the missing data values in our work. Furthermore, we compared several ML models with our model in the same dataset, which provided a better understanding of the performance. Additionally, we highlighted the shortcomings and flaws in the earlier heart disease prediction systems in this study. The type of data present in the dataset is one of the main factors for an ML model in previous studies to achieve the highest performance. Finally, the experiments showed that our proposed model achieved a comparative accuracy of 95.30% compared to the six other known methods from earlier studies. Furthermore, our approach still yields impressive outcomes even when limited to clinical data without employing the full feature set. This presents an opportunity for practitioners who lack access to laboratory tests or echocardiograms to make reliable diagnoses of heart failure without necessarily relying on supplementary tests. The proposed method can be widely applied and performs excellently with real-world data. In the future, we will experiment with our work with multiple combinations of machine learning models with extensive healthcare datasets. Additionally, larger data sets will make it possible for us to train deep neural networks efficiently. In our upcoming effort, we intend to gather institutional data and analyze research using various outlier detection techniques that should be explored further.

## Figures and Tables

**Table 1:** Key notations used throughout this article.

Complete Form	Short Form
Congestive heart failure	CHF
Cardiovascular health study	CHS
K-nearest neighbor	KNN
Machine learning	ML
Logistic regression	LR
Naive Bayes	NB
Random forest	RF
Support vector machine	SVM
Decision tree	DT
Deep neural network	DNN
World health organization	WHO
Electronic health records	EHRs
Synthetic minority over-sampling technique-edited	SMOTE
Synthetic minority over-sampling technique-edited nearest neighbor	SMOTE-ENN
Deep learning	DL
Logistic model tree	LMT
Leave-one-subject-out	LOSO
Principle component analysis	PCA
Hybrid random forest with linear model	HRFLM
Linear method	LM
Gaussian naive Bayes classifier	G-NB
Adaptive boosting	AdaBoost
Artificial intelligence	AI
Gradient boosting classifier	GBM
Stochastic gradient descent	SGD
Coronary heart disease	CHD
False positive rate	FPR
Matthews correlation coefficient	MCC
True positive	TP
False positive	FP
True negative	TN
False negative	FN

**Table 2:** A summary of all the prominent models for CHF prediction proposed in the last decade.

Sl. No.	Paper	Method	Dataset	Feature	Accuracy
1	Singh et al. [18]	Random forest	Cleveland heart disease dataset	303 instances, 14 attributes	85.81%
2	Gjoreski et al. [19]	ML, DL	UKC-JSI, PhysioNet, heart sounds	947 instances	92.9%
3	Plati et al. [20]	Rotation forest (ROT), logistic model tree (LMT)	University College Dublin, Ireland, and University Hospital of Ioannina	487 instances, 19 attributes	91.23%
4	Gjoreski et al. [21]	RF, naive Bayes, SVM, J48, KNN, bagging, and boosting	PCG sounds	152 instances	96%
5	Aljaaf et al. [22]	C4.5 Algorithm	Cleveland Heart Disease Dataset	297 instances, 13 attributes	95.5%
6	Srivastava et al. [23]	KNN	Cleveland Heart Disease Dataset	297 instances, 13 attributes	87%
7	Awan et al. [24]	PCA and ANN	the Cleveland UCI repository	297 instances, 13 attributes	97.7%
8	Mohan et al. [25]	HRFLM	The Cleveland UCI repository	297 instances, 13 attributes	88.7%
9	Shaji et al. [26]	ANN	Jubilee Mission Medical College and Research Institute Thrissur	2200 instances, 20 attributes	92.21%
10	Fitriyani et al. [29]	Hybrid SMOTE-ENN and XGBoost	Statlog and Cleveland dataset	270 instances, 13 attributes and 303 instances, 13 attributes respectively	95.90% and 98.40% respectively
11	Ishaq et al. [30]	Extra tree classifier	UCI machine learning repository	299 instances 13 attributes	92.62%

**Table 3:** List of selected features based on the gain ratio.

Sl. No.	Feature	Information	Data Type
1.	age	Age of subject	Numeric
2.	BMI	Body mass index	Numeric
3.	hyper	Calc. hypertension status	Numeric
4.	chstpn	Ever had pain in your chest	Categorical
5.	bp	High blood pressure	Categorical
6.	diab	Calc. diabetes status	Categorical
7.	smoke	Smoking status	Categorical
8.	exe	Exercise	Categorical
9.	weight	Weight	Numeric
10.	hrate	Heart rate	Categorical
11.	alcohol	Alcohol consumption	Categorical
12.	hibsug	High blood sugar status	Categorical

**Table 4:** A detailed description of the proposed architecture of DNN.

Parameter	Used
Input nodes	12
Hidden layers	4
Nodes of 1st hidden layer	50
Nodes of 2nd hidden layer	30
Nodes of 3rd hidden layer	20
Nodes of 4th hidden layer	10
Output nodes	1
Activation function	ReLU
Training method	SGD
Learning rate	0.001

**Table 5:** Hardware architecture of the system where the proposed model is trained and evaluated.

Hardware	Description
GPU memory	16 GB GDDR6 RTX-OPS 62T
Graphics bus PCI Express	3.0 × 16
CUDA parallel-processing cores	3072
FP32 performance	11.2 TFLOPS
NVIDIA RT cores	48
NVIDIA tensor cores	384

**Table 6:** Comparitive analysis of results obtained with different classifiers.

Classifier	Sensitivity	Specificity	Precision	FPR	Accuracy	F1-score	MCC	AUC
DT	0.8293	0.7188	0.9204	0.2813	0.8069	0.8725	0.4902	0.8234
NB	0.8421	0.7438	0.9279	0.2563	0.8221	0.8829	0.5269	0.8904
KNN	0.8533	0.7188	0.9224	0.2813	0.8259	0.8865	0.5229	0.8920
SVM	0.8772	0.7500	0.9322	0.2500	0.8513	0.9039	0.5827	0.8327
RF	0.9266	0.8188	0.9525	0.1813	0.9047	0.9394	0.7185	0.9352
LR	0.9490	0.8438	0.9529	0.1563	0.9276	0.9543	0.7803	0.9603
DNN	0.9649	0.9063	0.9758	0.0938	0.9530	0.9703	0.8575	0.9759

## Data Availability

The research data underlying this study will be made available by the corresponding author upon reasonable request. Access to the data will be provided in accordance with ethical and legal considerations, ensuring confidentiality and appropriate usage.
